# Report on Carbuncle

**Published:** 1855-04

**Authors:** 


*Report on Carbuncle.* From St. Bartholomew's Hospital.

The remarkable predisposition to furuncular and carbuncular inflammations hs been noticed by the American profession for the past two years, and although we are not aware of anything having been published on the subject wich has attracted much attention, yet the fact has been repeatedly recognied by the profession, and it has become quite a habit with us in meeting a nedical friend from different parts of the country, to inquire of him if he ad seen any whitiows or carbuncles.

The tendency to this particular form of inflammation which we have noiced, has been developed in an equal degree in England, and the medical urnals of that country have repeatedly called attention to the fact. A few xtracts from the Report* on the cases of carbuncle occurring in St. Bartolomew's during the last year will be not without interest to the reader.

*The recent increase of carbuncle* is plainly shown by this report. A refence to the book of the institution, gives the number of cases treated by M. Paget in the respective years—1842,–3,–4,–5,—None; whilst in the yer 1850 we have 8 cases; 1851, 4 cases; 1852, 5 cases, and 1853, 9 cases.

The Registrar General's report of the number of deaths from carbuncle in Ldon, furnish additional testimony. We find in 1841, 1 death—1842, 5—845, 9—1846, 3; whilst in 1852 there are registered 50 deaths; 1853, 70 and 1854 (up to November) 81 deaths from this disease. An accurate

---

* Med. Times and Gazette, December No.

table of thirty-five cases of carbuncle made out by the reporter from the books of the hospital, furnish the following data.

*The disease is found at all ages and under all circumstances*, and it is quite impossible to discover any hygienic cause; the robust and healthy are found together with the intemperate and cachectic; whilst some of the worst cases reported, have been brought from the healthiest parts of the country.

*Males are more liable to the disease than females.* Of the 35 cases, 29 were men and six women: the Registrar's report also corroborates this point. Of 49 deaths, the particulars of which are mentioned, 32 were men, 17 women. The same holds good with regard to furuncles. A calculation made from several hundred out patients of St. Bartholomew's gives the proportion as more than two to one.

*Diet* has but little influence on these cases; for in 33 cases, 12 were well fed, 10 moderately fed, 10 badly fed and 1 half starved.

*Part Effected.* No part of the body seems to be exempt from carbuncle. It is extremely rare on the scalp, and but one case has ever been reported where it appeared on the feet, (that case occurred to Mr. Coulson.) The favorite seats of carbuncle are those where the skin is the thickest. In the 25 cases reported, 12 appeared on the nape; 10 on the back, and 2 on the buttock.

*Treatment.* The general treatment was dependent very much on the symptoms and general condition of each case. High inflammatory fever being generally present in the beginning, was met with salines and low diet: opium was freely administered to control the pain and induce sleep, whilst quinine and ammonia would often be exhibited in the second stage with great effect. The local treatment at St. Bartholomew's was invariably to make free incisions into the carbuncle. An exception, however, was made in 8 cases of the 35 reported, which upon admission were doing well and free openings had been spontaneously formed. (These 8 cases go to prove that the incision is not always necessary.) The relief to pain, however, which follows the incision, is conclusive as to its propriety, even though the slough is larger than if it had been avoided. The fact that in 27 cases here reported, the progress of the disease was checked in 17 cases by the first incision, is satisfactory on this point.

The necessity of uniting the application of caustics with the incision, was not often recognized, though when the disease seems to spread itself with rapidity, they may be looked upon as indicated. Messrs. Startin and McWhinney at the hospital for Skin Diseases, prefer the nitrate acid of mercury; Mr. Ure, at St. Mary's, uses the potassa fusa for the same purpose.—*Ibid.*

